# Controlling the Morphology in Electrostatic Self-Assembly via Light

**DOI:** 10.3390/polym16010050

**Published:** 2023-12-22

**Authors:** Mohit Agarwal, Alexander Zika, Ralf Schweins, Franziska Gröhn

**Affiliations:** 1 Department of Chemistry and Pharmacy, Interdisciplinary Center for Molecular Materials, Friedrich-Alexander Universität Erlangen-Nürnberg, Egerlandstr. 3, D-91058 Erlangen, Germany; 2 Institut Laue-Langevin, DS/LSS, 71 Avenue des Martyrs, F-38000 Grenoble, France; schweins@ill.eu

**Keywords:** electrostatic assembly, light irradiation, nanostructures, polyelectrolyte, thermodynamically controlled, kinetically controlled

## Abstract

Electrostatic self-assembly of macroions is an emerging area with great potential in the development of nanoscale functional objects, where photo-irradiation responsiveness can either elevate or suppress the self-assembly. The ability to control the size and shape of macroion assemblies would greatly facilitate the fabrication of desired nano-objects that can be harnessed in various applications such as catalysis, drug delivery, bio-sensors, and actuators. Here, we demonstrate that a polyelectrolyte with a size of 5 nm and multivalent counterions with a size of 1 nm can produce well-defined nanostructures ranging in size from 10–1000 nm in an aqueous environment by utilizing the concept of electrostatic self-assembly and other intermolecular non-covalent interactions including dipole–dipole interactions. The *pH*- and photoresponsiveness of polyelectrolytes and azo dyes provide diverse parameters to tune the nanostructures. Our findings demonstrate a facile approach to fabricating and manipulating self-assembled nanoparticles using light and neutron scattering techniques.

## 1. Introduction

Self-assembly generates complex structures with a variety of functional properties over a range of length scales. Diverse structures such as cell membranes [1,2], supramolecules [3,4,5], and protein complexes [6,7,8,9,10] are formed via multiple non-covalent interactions between adjacent molecules that occur due to their chemical nature. Recently, electrostatic self-assembly has emerged as one of the most facile approaches for fabricating such synthetic particles in aqueous solution [11,12,13,14]. This process is mainly driven by thermodynamics [15,16]; however, kinetics is also found to be an essential factor in structural manipulation [17]. Non-covalent interactions determine the lowest energy state that provides thermodynamic control and eventually stabilized nanostructures. Due to their relatively weak nature, these non-covalent interactions can be altered using various parameters, including pH, concentration, preparation method, charge ratio, and counterions. The precise manipulation of the nano-objects with external stimuli is critical to multiple applications ranging from nanoelectronics [11,13,18,19,20,21] to controlled drug delivery [22,23,24]. Light responsiveness as a non-invasive trigger attracted the most attention in this respect [14,25,26]. An additional advantage of this external stimulus is that it can be applied and switched back ‘on demand’ because of the excellent reversibility of the interactions. For example, light irradiation has been used to control a nanoparticle’s size and shape [11,14,25,26,27], giving rise to diverse functions and applications, including therapy [28,29,30] and sensors [31,32,33].

In electrostatic self-assembly, nanoparticle formations are fundamentally based on the nature of charges involved within the structure [11]. These charges arrange themselves to form assembled structures [34]. Generally, the electrostatic interactions are non-directional, and thus are best combined with a second kind of interactions such as dipole–dipole, π–π bonding, hydrogen bonding, hydrophobic effects, and van der Waals forces that provide the directionality for the structural formation [16,17,35,36,37,38]. Often, short-range secondary interactions are induced when long-range electrostatic forces bring building blocks together. Recent studies have been performed to understand the role of charges using different parameters such as concentration and charge ratio, *l_c_* (the ratio of the number of anionic charges to the number of cationic charges multiplied by the concentration of their anions and cations, respectively) [13,14,39]. Mariani et al. used a cationic polyamidoamine (PAMAM) dendrimer of different generations (2–8) for well-defined cationic macroions and mono-, di-, and trivalent azo dyes as anionic molecular counterions as a model system, where they observed the effect of generation dependency on the shape of the assemblies. The nano-assemblies transformed from isotropic to anisotropic structures when increasing the dendrimer generations [17].

Moreover, divalent azo dyes such as Acid Yellow 38 (AY38) and Direct Yellow 12 (DY12) have also been used recently in electrostatic assemblies to introduce photosensitivity in the system [14,25,26]. The negatively charged azo dyes bind with the dendrimer molecules in neutral and acidic mediums. These azo dyes can then isomerize from *trans* state (thermodynamically favored) to *cis* form (less favored) upon UV irradiation. In the case of AY38, the half-life (t_1/2_) of the *cis* state is approximately 12 h at pH = 3.5 (t_1/2_ ≈ 5 h for DY12) [25,40,41]. Moldenhauer et al. used the photoresponsive behavior of AY38 with linear polyelectrolytes to study the electrostatic assemblies [42]. Previously, electrostatic assemblies have been formed using AY38 dye and G4 PAMAM dendrimer by Willerich et al. [14], where the pH control and light irradiation caused reproducible nano assemblies. Later, Zika et al. [13] introduced the effect of photoacids in this type of system and created multi-switchable electrostatic assemblies with defined sizes and shapes.

**Scheme 1:**
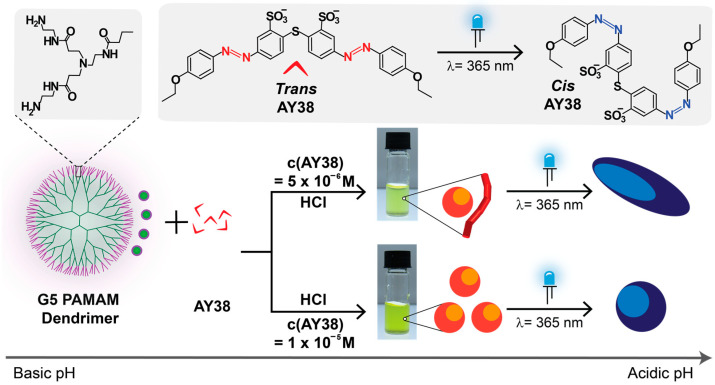
Preparation of electrostatic self-assembly of AY38 azo dye and G5 PAMAM dendrimer and effect of building blocks concentrations on the nanoparticle structures in aqueous solution.

In the present work (Scheme 1), we used the G5 PAMAM dendrimer, which has almost twice the positive charges of G4 and is more rigid than lower generations. Generation 5 PAMAM dendrimer comprises 128 primary and 126 tertiary amines that do not show any charges in the basic medium (at pH = 10.5). However, on decreasing the pH, primary amines become protonated and cationic, and finally, at pH = 3.5, all the amine groups (254) become positively charged. Generation 5 is also slightly larger in size (5.4 nm) as compared to G4 PAMAM dendrimer (4.5 nm) [43].

Herein, we aim to investigate the shape of the assemblies before and after irradiation, understand the kinetic and thermodynamic aspects of electrostatic assemblies as well as factors that lead to monodisperse versus multimodal assemblies, and attempt to provide an understanding that allows controlling these factors. Combining the effects of the charge ratio and photoswitchability on shape transformation has not yet been explored, to our knowledge. Here, we used these two parameters to tune the size of nanoparticles over a vast range of length scales (10 nm to 3 μm) with various configurations such as spheres, cylinders, flexible cylinders, and ellipsoids. To gain a more insightful understanding of the mentioned prospects, the thorough characterization of the nanoparticles will be demonstrated and discussed in the following.

## 2. Materials and Methods

### 2.1. Chemicals

Generation 5 PAMAM dendrimer was purchased from Aldrich, Darmstadt, Germany, and dynamic light scattering (DLS) in D_2_O and H_2_O confirmed the size (5.4 nm) given by the manufacturer Dendritech, Midland, MI, USA. AY38 (>40% purity) was purchased from Merck, Darmstadt, Germany, and purified to > 98% by multiple recrystallizations from water and ethanol, as described previously [42,44]. D_2_O, NaOD, and DCl were purchased from Merck, Darmstadt, Germany, and NaOD and DCl were used to adjust the *pD* of the solution.

### 2.2. Sample Preparation

As small-angle neutron scattering (SANS) is central to this study, we used D_2_O as a solvent and all regents in the deuterated form except for the assembly components throughout the complete study. All samples were prepared using D_2_O as a solvent in the basic medium at *pD* = 10.5. The azo dye stock solution (1.4 × 10^−3^ mol L^−1^) was diluted with D_2_O depending on the charge ratio. The appropriate amount of dendrimer stock solution (4.0 × 10^−5^ mol L^−1^) was added under continuous stirring for ideal mixing. Later, a reasonable amount of standard 0.1 mol L^−1^ DCl solution (until *pD* = 3.5) was added in one shot while stirring to induce the assembly formation. The *pH* measurements were performed using a glass electrode on a Mettler Toledo *pH* meter. The *pH* obtained from the *pH* meter was converted into *pD* values according to [45,46,47]:

(1) pD=pH+0.41

The sample was irradiated in a vial while stirring using a UV lamp at 365 nm wavelength for 20 min. A longer irradiation time did not influence the results, suggesting that the azo dye’s photostationary state was reached. Measurements were performed shortly after irradiation.

### 2.3. Light Scattering

Static and dynamic light scattering (SLS, DLS) are highly suitable techniques for characterizing polyelectrolytes in solution. The DLS, also known as photon correlation spectroscopy (PCS), investigates the fluctuation in the scattered intensity with time (Brownian motion of the scattering particles). This technique exploits quasi-elastic scattering and probes temporal fluctuations of the scattered intensity by using coherent laser light. Instead of using the scattering angle *θ*, the scattering vector $\vec{q}$ (also known as momentum transfer) is defined as $\vec{q}=\vec{K_{s}}-\vec{K_{i}}$, where $\vec{K_{s}}$ and $\vec{K_{i}}$ are the propagation vectors of scattering and incident light, respectively. The magnitude of *q* can be given by:

(2) $$q=\left|\vec{q}\right|=\frac{4\pi n_{D}}{\lambda }sin\left(\frac{\theta }{2}\right)$$

with *λ* being the wavelength of incident light in solution.

The intensity–time autocorrelation function, *g*_2_(*q*,*τ*) in DLS is measured by:

(3) $$g_{2}\left(q,\tau \right)=\frac{\langle I\left(q,t\right)I\left(q,t+\tau \right)\rangle }{{\langle I\left(q,t\right)\rangle }^{2}}$$

The above function describes the correlation between the scattering intensity at time *t* = 0 to time t + *τ*, where q is the momentum transfer and *τ* is the shift in time. The Siegert relation can convert the intensity–time autocorrelation function into field–time autocorrelation function g_1_(*q*, *τ*):

(4) $$g_{2}\left(q,\tau \right)=1+{\left[g_{1}\left(q,\tau \right)\right]}^{2}$$

The *g*_1_(*τ*) was analyzed by a regularized inverse Laplace transformation using the CONTIN algorithm by S. Provencher [48,49] to obtain the intensity-weighted relaxation times distribution. Mean relaxation times were then transferred into apparent diffusion coefficient *D_app_* using the relation:

*D_app_ = q^−2^τ^−1^* (5)

Measurements covered a broad range of *q* by measuring a scattering angle of 20° ≤ θ ≤ 150° with a 5-degree step. To measure the hydrodynamic radius (*R_H_*), *D_app_* extrapolated to *q* = 0. From this, the *R_H_* can be calculated using the Stokes-Einstein relationship:

(6) $$D\left(q^{2}=0\right)=\frac{kT}{6\pi {\eta R}_{H}}$$

where *k* is the Boltzmann constant, *T* is the absolute temperature in Kelvin, and *η* is the solvent viscosity.

The macroscopic scattering cross-section $\frac{d\sum }{d\Omega }\;$*(q)* for light scattering is commonly denoted as the Rayleigh ratio ΔR_θ_:

(7) $$\frac{d\sum }{d\Omega }\left(q\right)=\Delta R_{\theta }=KcM_{w}P\left(q\right)$$

where *K* is the optical constant, *c* is the mass concentration, *M_w_* is the weight-averaged molecular weight, and *P*(*q*) is the form factor. *K* is given by:

(8) $$K=\frac{4\pi^{2}}{\lambda_{0}^{4}N_{A}}{\left(n_{D,0}\frac{dn}{dc}\right)}^{2}$$

with λ_0_ being the wavelength of the incoming light, *N_A_* the Avogadro constant, *n_D_*_,0_ the refractive index of the standard solution used to calibrate absolute intensity, and *dn*/*dc* the refractive index increment. Either Guinier approximation can be used to determine the size via *R_G_* at low *q*:

(9) $$P(q)\approx exp\left(\frac{-q^{2}R_{G}^{2}}{3}\right)$$

or the Zimm equation:

(10) $$\frac{Kc}{∆R_{\theta }}=\frac{1}{M_{w}}\left(1+\frac{1}{3}q^{2}\langle R_{G}^{2}\rangle \right)+2A_{2}c$$

where *R_G_* is the radius of gyration, *A_2_* is the second osmotic virial coefficient, and *c* is the mass concentration.

The ratio of *R_G_*/*R_H_*, also known as the 𝜌 ratio, provides essential information on the scattering particle topology, especially in the small range of particles (size = 10–100 nm) due to the limited length scale of light scattering [50]. SLS data have also been combined with small-angle neutron scattering (SANS) data to extend the low *q*-range for large aggregates. Combined light scattering techniques give primary hints on the particle shape, while small-angle scattering is needed for a thorough structural analysis.

Measurements were carried out using an ALV CGS3 goniometer with an ALV 5004 correlator (ALV GmbH, Langen, Germany) equipped with a HeNe laser with a wavelength of *λ* = 632.8 nm and a maximum output power of 23 mW.

### 2.4. ζ-Potential

The particle and its microenvironment determine the electrostatic properties of nanostructures in a solution. Such properties of nanostructures can be characterized by their *ζ*-potential measurements. This technique measures the difference in electrostatic potential between the medium and the fluid layer of dispersed nanostructures. The *ζ*-potential was calculated from the electrophoretic mobility using the Helmholtz–Smoluchowski equation below [51,52]:

(11) ζ=(μ∗η)/ε

where *μ* is the electrophoretic mobility of charged particles, *η* is the viscosity of the solution, and *ε* is the dielectric constant of the dispersive medium.

The *ζ*-potential measurements were conducted on a Zetasizer Nano ZS analyzer with an integrated 4 mW HeNe laser, *λ* = 633 nm (Malvern Instruments Ltd., Malvern, UK).

### 2.5. UV-Vis Spectroscopy

Absorption spectra were recorded on a JASCO V-630 spectrometer using 1 mm pathlength quartz cuvettes. Room temperature was used for each measurement. For the reference measurements, D_2_O was used as a solvent. UV-Vis measurements were also performed to calculate the amount of *trans* and *cis* isomers in the solution and in the assemblies. The fraction of the bound dye molecules was calculated by the following formula:

(12) $$Fraction\;of\;bound\;Cis\;dye=\frac{quantity\;of\;Cis\;dye\;in\;the\;assemblies}{total\;quantity\;of\;dye\;in\;the\;assemblies}$$

(13) $$=\frac{Cis\;area\left(before\;centr.\right)-Cis\;area(supernat.)}{\left(Cis\;area\left(before\;centr.\right)-Cis\;area\left(supernat.\right)\right)+(Trans\;area\left(before\;centr.\right)-Trans\;area(supernat.))}$$

The value obtained from the above formula was multiplied by 100 to find the bound *cis* % in the assemblies. The area of the isomers was calculated using the Gaussian model in Origin 9.0 software as follows:

(14) $$y=y_{0}+(A/(w\sqrt{\pi /2}))e^{\left(-2({\left(x-xc\right)/w}^{2})\right)}$$

where *y* is the Gaussian function, *y*_0_ is the offset, *xc* is the center of the peak, *w* is the width, and *A* is the area of the peak.

### 2.6. Small-Angle Neutron Scattering

As the scattering length of neutrons (b) depends on the isotope and is significantly different for hydrogen and deuterium, using deuterated compounds or solvents to get higher contrast using neutrons is advantageous to analyzing organic material in solution. SANS measurements were performed at beamline D11 at the Institut Laue–Langevin (ILL), Grenoble, France. The proposal numbers of the beamtimes were 9-12-662 [doi:10.5291/ILL-DATA.9-12-662], and 9-12-624 [doi:10.5291/ILL-DATA.9-12-624].

SANS samples were prepared in D_2_O with mainly two different azo dye concentrations of c_AY38_ = 0.5 × 10^−4^ mol L^−1^ and 1.0 × 10^−4^ mol L^−1^ and transferred into Hellma 404-QX quartz cells with a 2 mm path length. The employed neutron beam was 15 mm in diameter. At neutron wavelength *λ* = 6.0 Å (FWHM 10%), three different sample–detector distances (1.7, 8.0, and 38 m) were used with a collimation distance of 4.0, 8.0, and 40.5 m to cover the whole particle. A total scattering vector range of 0.016 nm^−1^ ≤ *q* ≤ 4.9 nm^−1^ was investigated. Scattered neutrons were detected using a multitube ^3^He gas detector consisting of three detector panels: a central one with 192 horizontally oriented tubes, and a left- and right-side panel of 32 vertically oriented tubes. Each tube was 1 m long with a pixel size of 4 mm along the tube, with the inner tube diameter being 8 mm. The detector was, therefore, composed of 256 × 256 pixels. Two-dimensional data were corrected for empty cell scattering, electronic background, detector uniformity via measuring 1 mm H_2_O scattering, and transmissions using the Mantid 6.0 software package [53]. Data were put on an absolute scale via the attenuated direct beam intensity, measured per instrument configuration. Azimuthally averaged scattering curves were then used to subtract the solvent scattering and incoherent background.

The SANS intensity after data correction is:

(15) $$I\left(q\right)=\emptyset V_{NP}{\left({∆\rho }_{SLD}\right)}^{2}P\left(q\right)S(q)$$

where *∅* is the volume fraction, *V_NP_* is the particle volume, ${∆\rho }_{SLD}$ is the difference in scattering length densities between the solute and the solvent, and *P*(*q*), the particle form factor, corresponds to the particle shape. The structure factor *S*(*q*) represents the interparticle correlations considered equal to 1, as the samples were diluted.

For example, for a sphere with radius *R*, the form factor *P*(*q*) is [54]

(16) $$P\left(q,\alpha \right)=\frac{scale}{V}{\left(3\frac{sinqR-qRcosqR}{{\left(qR\right)}^{3}}\right)}^{2}+background$$

while for a cylinder with length *L* and radius *R*, *P*(*q*) is [55,56]

(17) $$P\left(q,\alpha \right)=\frac{scale}{V}{\left(2\left(∆\rho \right)V\frac{sin\left(\frac{1}{2}qLcos\alpha \right)}{\frac{1}{2}qLcos\alpha }\frac{J_{1}\left(qRsin\alpha \right)}{qRsin\alpha }\right)}^{2}+background$$

where *α* is the angle between the cylinder’s axes, *V* = πR^2^L is the volume of the cylinder, Δ*ρ* (contrast) is the scattering length density difference between the scatterer and the solvent, and *J*_1_ is the first-order Bessel function.

For an ellipsoid, the form factor *P*(*q*) is:

(18) $$P\left(q,\alpha \right)=\frac{scale}{V}{\left(∆\rho V\frac{3(\sin qr-qr\;cos\;qr)}{{\left(qr\right)}^{3}}\right)}^{2}+background$$

with

(19) $$r={\left[R_{e}^{2}{sin}^{2}\alpha +R_{p}^{2}cos\alpha \right]}^{1/2}$$

where *α* is the angle between the ellipsoidal axes and $V=(4/3)\pi R_{p}R_{e}^{2}$ is the volume of the ellipsoid, *R_p_* is the polar radius along the rotational axis, and *R_e_* is the equatorial radius perpendicular to the rotational axis of the ellipsoid.

As explained above in SLS, the Guinier radius can also be used for low-q SANS data, where ln(q) is plotted against q^2^ [54]. Guinier radius is usually restricted to small-q ranges only until the Guinier law follows (q^2^ × R_g_ ≤ 1.2). Therefore, it is applied only for small-sized nanoparticles (1–100 nm).

SasView 4.2.2 was employed to fit the scattering data using various model form factors. The reduced χ^2^ value can assess the SANS data’s fitting quality. The polydispersity index (PDI) is also provided using this software.

### 2.7. Isothermal Titration Calorimetry

ITC experiments were performed at the VP-ITC microcalorimeter from MicroCal Inc. (Northampton, MA, USA).

ITC is a technique that measures the heat changes (released or absorbed) upon mixing two solutions. Two identical reference and sample cells with a volume of 1.442 mL each are enclosed in an adiabatic jacket. In this study, the reference cell was filled with the solvent (D_2_O), and the working cell contained the sample solution. A volume of 288 μL of titrant from the syringe was injected stepwise into the sample cell under constant stirring. For the presented experiments, the stirring rate was 351 rpm. All the measurements were performed at RT (25 °C). Small aliquots of titrant (typically 2 μL) were injected in 50–60 steps to achieve the charge ratio range of 0.2 ≤ *I_c_* ≤ 4.4. The duration of each injection was 300 s with a total measurement time of 4–4.5 h (including equilibration time). Due to the absorption or release of heat, each injection produces a distinctive peak. A separate measurement was performed with the titrant and the solvent for the data subtraction. The data analysis and fitting were performed using the Origin 7.0 software provided by MicroCal. The data were fitted with the two-site model. Gibbs free energy (Δ*G*) was calculated using a formula:

(20) ∆G=∆H−T∆S=−RTlnK

## 3. Results

The present study focuses on the physical–chemical aspects of the formation of nanoparticles in solution with defined sizes and varying shapes formed by electrostatic self-assembly of acid yellow 38 azo dye (AY38) and generation 5 PAMAM dendrimers. Here, the effect of irradiation has been studied while varying the charge ratio and concentration of these assemblies. In this study, the charge ratio, *l_c_*, is represented as the ratio of the number of anionic dye sulfonates groups (originating from AY38) to the number of cationic amine groups (primary and tertiary amines originating from PAMAM dendrimer). Due to the different number of ions in both building blocks (AY38 has two anions, and G5 dendrimer has 254 cations), a higher dye concentration was used to balance the charge ratio.

Due to the pH responsiveness of PAMAM dendrimers, stock solutions of dye and dendrimer were mixed in water in a basic medium (*pD* = 10.5) to prevent assembly formation. Later, to initiate the self-assembly process, a small amount of DCl was added until the *pD* of the solution declined to *pD* = 3.5. These nanoparticles were characterized by various methods, as will be discussed in the following:

### 3.1. Light and Small-Angle Neutron Scattering

**Figure 1:**
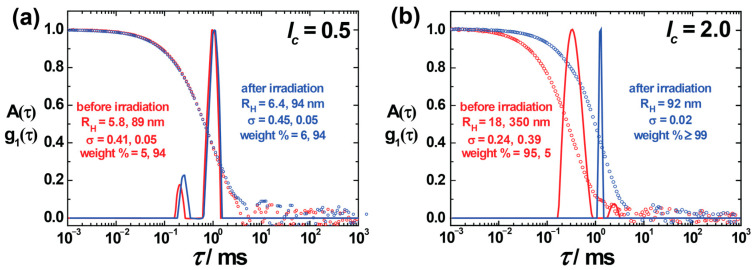
Dynamic light scattering on AY38/G5 assemblies at charge ratio (**a**) *l_c_* = 0.5 and (**b**) *l_c_* = 2.0: Electric field autocorrelation function g_1_(τ) and distribution of relaxation times A(τ) at a scattering angle of θ = 90 show the increase in assembly size. R_H_ = hydrodynamic radius; σ = standard deviation (peak width) of the distribution.

Light scattering experiments were performed for the nanoparticle size evaluation, where the hydrodynamic radii R_H_ were received from DLS, and SLS was performed to obtain information on scattered particle topology. The ratio of the radius of gyration, R_G_ (obtained from SLS) to R_H_, gives an indication of the nanoparticles’ shape before analyzing it in detail by SANS. For AY38/G5 assemblies with two charge ratios, *l_c_* = 0.5 and *l_c_* = 2.0, the electric field autocorrelation functions g_1_ (τ) are plotted together with the corresponding relaxation time distributions in Figure 1. DLS indicated stable assemblies of different sizes at different charge ratios. A bimodal distribution with hydrodynamic radius, R_H_ = (5.8 ± 2.4) nm and R_H_ = (89 ± 4.5) nm, was observed (red color) at *l_c_* = 0.5 (Figure 1a). The small-size peak (intensity weight % = 5%) originates from excess free dendrimers in the solution. Irradiation does not have any major effect on the aggregates in this case. At *l_c_* = 2.0, again two different types of aggregates form, with R_H_ = (18 ± 4) nm and R_H_ = (350 ± 140) nm. According to DLS, the primary peak (intensity-weighted) contributes to the smaller particles (≈ 95%) prior to irradiation, which shows that the majority of particles are small particles in the solution compared to large aggregates. An enormous change in particle size (up to five-fold) was noticed upon irradiation, with a significant decrease in distribution width (σ ≈ 0.24 to 0.02), as can be seen in Figure 1b. The results agree with the previous studies, where, however, polydispersity and bimodal distributions were not explored in detail [42,57].

The charge ratio has been one of the critical factors in altering the electrostatic assemblies in our recent studies [14,16]. However, photoswitchability and its effects on particle morphology have yet to be fundamentally understood in detail.

**Figure 2:**
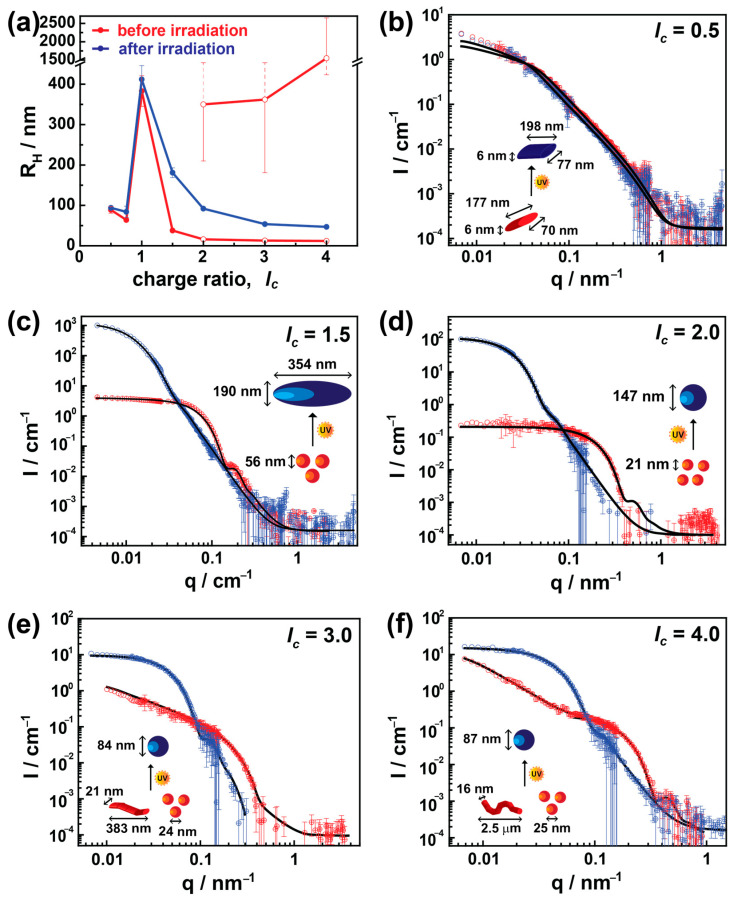
The effect of charge ratio and irradiation on AY38/G5 photosensitive assembly: (**a**) hydrodynamic radius, R_H_, obtained by multiangle DLS (open red symbols for *l_c_ =* 2.0, 3.0, and 4.0 indicate a bimodal distribution); (**b**–**f**) SLS-SANS. Red and blue color represent the nanoparticles before and after irradiation. Black lines represent the best structural model fit. The inset sketches also indicate corresponding shapes.

The DLS results are displayed in Figure 2a, where the hydrodynamic radius, R_H_, is plotted against *l_c_*. The red and blue colors in Figure 2 represent assemblies before and after irradiation, respectively. In excess of the dendrimer (*l_c_* = 0.5 and 0.75), nearly monomodal particles with some free G5 PAMAM dendrimers were observed in the solution. At *l_c_* = 1.0, the building blocks form highly aggregated and large nanostructures (up to 1 μm diameter) and will be discussed later in conjunction with the ζ-potential studies.

Upon increasing the *l_c_* to moderate excess of dye (*l_c_* = 1.5), nearly monodisperse particles form with the size of R_H_ = 19 nm, and the previously unbound dendrimer molecules now associate with the assemblies. On further increasing the dye content (*l_c_* ≥ 2.0), a bimodal distribution (a mixture of 10–20 nm nanoparticles and large aggregates up to micrometer sizes) with increased polydispersity appears. These sizes are represented by the open symbols along with their charge ratios. Only the 5% intensity weight contribution comes from the larger aggregates at *l_c_* = 2.0, which increases to 30% and 40% at *l_c_* = 3.0 and 4.0, respectively (Figure S1). Upon expanding the *l_c_* to a very high excess of dye (*l_c_* = 30.0), only 5% of the intensity-weighted particles were found to be small particles (R_H_ = 12 nm); otherwise, the rest of them form large aggregates up to 200 nm radius as shown in Figure S2. This suggests that the smaller assemblies are similar to the ones observed at *l_c_* = 1.5. With increasing amounts of dye, the interaction increases between these assemblies, and larger aggregates are formed. These large aggregates could be the result of a kinetic process. Irradiating these assemblies using λ = 365 nm significantly modulates them depending on their charge ratios. No notable changes in size have been observed at *l_c_* ≤ 1.0, where the cationic charges from dendrimers stabilize the charged nanostructures. Thus, the isomerization of the dye from *trans* to *cis* does not influence the stability of the nano-assemblies, and the size remains the same [14].

On increasing the charge ratio, i.e., where the anionic charges dominate, the polydispersity decreases sharply upon irradiation, and the light restructures these bimodal aggregates to produce well-defined monodisperse nanoparticles with a size between R_H_ = 47 nm and R_H_ = 181 nm depending on their charge ratio. The most substantial difference in size occurred at a moderate excess of dye (*l_c_* =1.5), whereas for larger charge ratios, the size increment is not as immense as in *l_c_* = 1.5. In addition, at higher *l_c_*, the larger and smaller aggregates (bimodal system) dissolve to fabricate monomodal nanoparticles upon irradiation.

Along with the charge ratio, the concentration effects were also observed in the case of a high excess of dendrimer (*l_c_* < 1.0) and a moderate excess of dye charges (1.0 ≤ *l_c_* ≤ 2.0). The DLS result of these charge ratios in Figure S3 shows the effect of concentration and irradiation on the assembly sizes. The size decreases with the increase in concentration. It must be due to the absolute amount of excess charges (anionic or cationic) in the assemblies that help stabilize the assemblies and inhibit the growth of aggregates.

To sum up the results from light scattering, it was observed that highly monodispersed assemblies form upon irradiation, and the largest change in size is observed in the case of moderate excess dyes. With increasing the charge ratio and concentration, the surplus dye ions tend to stabilize the assemblies, which might be a reason for small changes in size due to irradiation, as previously explained by Willerich et al. [14]. Moreover, in the excess of dye ions, secondary dye–dye interactions take place, which could be the reason for polydispersity in the samples. The role of charged species regarding stability and shape modifications are further investigated in detail in ζ-potential and UV-Vis studies mentioned below.

After characterizing these nanoparticles in detail using light scattering, it is highly interesting to investigate the detailed shape of the particles. Therefore, the photo and charge ratio effects on the nanoparticle structures, particularly the shape, have been addressed by combining results from static light scattering and small-angle neutron scattering measurements, as shown in Figure 2b–f. SLS data merged with SANS data very well in all cases. The Guinier radii corresponding to the structural model fits are well in agreement with the Guinier radii obtained from Guinier plots, which again confirms the model’s accuracy (Table S1).

Figure 2b shows data for *l_c_* = 0.5. Data are fitted using an elliptically elongated cylinder model with a length of (177 ± 22) nm and an elliptical cross-section of an axial radius of 3 nm, that is, well-defined elongated particles with a remarkable axis ratio of 13. No significant structural changes occur after irradiation; the shape remained with a similar radius and axis ratio, exhibiting a length of (198 ± 35) nm after irradiation. The results are in agreement with DLS. The excess of positive charges from dendrimer molecules provides stability to the assemblies, and due to the decreased amount of azo dye, no changes are observed upon irradiation.

Contrarily, a substantial difference in size and shape has been observed in the case of a moderate excess of dye (*l_c_* = 1.5). Here, small spherically shaped nanoparticles of diameter (56 ± 10) nm convert to ellipsoidal assemblies with an axial length of (190 ± 16) nm and equatorial length of (395 ± 40) nm with a low polydispersity (σ ≤ 0.10) due to irradiation, as shown in Figure 2c. In the case of *l_c_* = 2.0, again, a considerable difference in size has been observed after irradiation, where 21 nm nanoparticles are converted into 147 nm spherical particles. However, prior to irradiation, a few data points (obtained from SLS measurements) at low-q do not fall within the range of a quality fit. This is possibly due to a small number of large aggregates observed in DLS (R_H_ = 350 ± 140 nm) and the limited length scale of SANS.

On further increasing the charge ratio (*l_c_* > 2), bimodal particle distributions with different particle shapes and large polydispersity are observed. These coexisting shapes were small spherical objects (20–40 nm) along with exceedingly elongated flexible or elliptical cylinders with a length of up to μm sizes, where the small aggregates are in excess (>80%). Again, this agrees with the light scattering results. As the bimodal assemblies begin to be seen at an excess of dye (*l_c_* ≥ 2.0), this may indicate that secondary interactions between dye molecules must play a pivotal role in inducing the polydispersity. Interestingly, upon irradiation, even though there are two highly different structural nano-assemblies, these bimodal assemblies transform into highly monodisperse spherical particles. This can be understood with the isomerization of the azo dye to the *cis* state. Due thereto, the surplus dye ions associated with secondary non-covalent interactions start to disassemble. Thus, monodispersed nano-assemblies are formed, similar to the previous study by Willerich et al. [14]. More details will be given in ζ-potential and UV-Vis studies below.

After observing the concentration effect on the assembly size using light scattering, it is further explored in SANS (Figure S4) using various charge ratios and 0.5 × 10^−4^ mol L^−1^ concentration (in terms of AY38). Before irradiation, charge ratios *l_c_* = 2.0, 3.0, and 4.0 show a similar bimodal distribution with small spherical aggregates and elongated micrometer-sized structures. In this case, the smaller aggregates were larger (≈36 nm) than those observed at higher concentrations (≤25 nm) for the same charge ratios. Various factors could be involved in controlling the size of the assemblies by varying the concentrations. At lower concentrations, the number of dye-loaded dendrimers formed in the beginning during the self-assembly process decreases, leading to a higher number of building blocks being available for the nano-assembly growth, resulting in large aggregates [58]. As the concentration increases, more dye-loaded dendrimers form, competing for the free building blocks. This restricts the growth of each particle, leading to smaller aggregates. The saturation level of dye–dendrimer and dye–dye interactions is achieved at higher concentrations, so further concentration increases do not significantly affect the size [59,60,61]. Moreover, at lower concentrations, the electrostatic repulsion between the dye-loaded dendrimers decreases due to large interparticle distances, which allows them to interact and form larger assemblies, whereas in higher concentrations, due to high electrostatic repulsion, compact assemblies form. At lower concentrations, due to the decreased amount of dye and dendrimer molecules, the particles likely have a longer diffusion time before encountering other molecules. This extended diffusion time can result in a lower amount of dye–dye interactions, which leads to less stabilized nano-assemblies and the particles growing in size.

After irradiation, highly monodisperse (PDI ≤ 0.2) spherical structures are formed from the bimodal distribution. Compared to the higher concentration, the size of the nano-assemblies is larger, similar to before irradiation. This can be understood with the larger size of the nano-assemblies before irradiation.

All the structures obtained from SANS before and after irradiation, including concentration and charge ratio effects, are summarized in Table 1. The size is not up to the scale in the table. For low concentration, the data are not available for *l_c_* = 0.5 and 1.5. Upon irradiation, the shape of the assemblies becomes more isotropic when the charge ratio increases, irrespective of their concentrations. Thus, evidently, a decreased dye–dye interaction causes the formation of more isotropic aggregates, as found previously for non-switchable systems [39]. This is also accompanied by a lower surface charge density (charged dye molecules detach), for which the smaller surface to be stabilized of the more isotropic structure likely is favored.

### 3.2. Kinetic and Thermodynamic Assemblies

Well-defined self-assembled structures can be thermodynamically controlled, i.e., represent equilibrium structures, or kinetically controlled, i.e., be kinetically trapped, as due to a high activation energy, the thermodynamic minimum is not reached [17,62,63]. The preparation route can thus affect the outcome of a kinetically controlled particle design, i.e., by various reaction conditions, including temperature, order of mixing, and stirring velocity. In contrast, more stable assemblies and the lowest free energy, thus independent of the preparation route, favor the thermodynamically controlled structure. According to the previous studies, assemblies dissolve again upon adding excess dendrimer, for example, when changing the charge ratio from *l_c_* = 0.75 to *l_c_* = 0.5, which suggests that the nanostructures are not kinetically trapped but adjust to the respective thermodynamically favored state for each composition ratio [39]. From this, it can be deduced that the nanoparticles and building blocks are in equilibrium.

As the above experiment was qualitative, a detailed study of different mixing procedures is needed to gain a deeper insight into the assembly formation in solution. Since the assembly formation upon mixing the charged components takes place on a fast time scale, separating the mixing steps of building blocks would be essential to understanding the mechanics of assembly formation. In the standard sample preparation method I (developed from previous studies), both the components are first mixed at *pD* = 10.5, and subsequent adjustment to *pD* = 3.5 follows, which offers the advantage of a complete mixing prior to assembly formation due to deprotonated dendrimer being uncharged [14,25,39]. In contrast, in methods II and III, the stock solutions were prepared in acidic medium (*pD* = 3.5). The dendrimer molecules were entirely charged when mixed with an acidic dye solution (the dendrimer solution was incorporated into the dye solution in method II and in the opposite way in method III).

**Figure 3:**
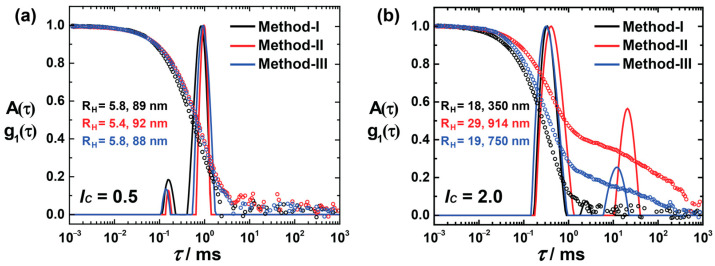
DLS for different preparation routes: Electric field autocorrelation function g_1_(τ) and distribution of relaxation times A(τ) (scattering angle θ = 90°) of AY38/G5 assemblies before irradiation: (**a**) at *l_c_* = 0.5 and (**b**) at *l_c_* = 2.0, where method I is the preparation way used throughout this study. In methods II and III, stock solutions were prepared and mixed in acidic medium (*pD* = 3.5). In method II, the dendrimer solution was added to the dye solution, while in method III, it was the other way around.

Figure 3a shows the size distribution of *l_c_* = 0.5, prepared using methods I, II, and III. All the assemblies show bimodal distributions, where the small aggregates must originate from the excess of dendrimer molecules, and the large aggregates are the product of dye–dendrimer interactions. Irrespective of preparation routes, all the assemblies are similar in size, which suggests that these assemblies are thermodynamically controlled. At *l_c_* = 2.0, diverse structures are formed based on their methods of preparation (Figure 3b), whereas at *l_c_* = 2.0, larger aggregates result (along with the small assemblies) with a significant standard deviation and a large quantity of the assemblies. The hydrodynamic radius of the small aggregates is in the range of 18–30 nm_._ However, the larger aggregates vary, and methods II and III form aggregates with R_H_ > 500 nm_._ Thus, the nano-assemblies—to be more exact, the larger nanoparticles—are kinetically controlled when there is an excess of anionic charges.

After irradiation, light-scattering results were observed, as given in Figure S5, for both charge ratios. No significant changes in size occur at *l_c_* = 0.5, but larger particles are observed using methods II and III in a higher charge ratio. All the large aggregates detected before irradiation disassemble and fabricate more monodispersed assemblies with small particles. Using SANS, Figure S6 reveals the structure variation (size and shape) by changing the preparation route for *l_c_* = 2.0. SANS reveals the elongated structures prior to irradiation. These elongated particles and smaller spheres reassemble to form highly monodispersed spheres upon irradiation, as addressed above.

In general, this kinetic influence appears only in high charge ratio (*l_c_* > 1.5) and before irradiation. Until *l_c_* ≤ 1.5 and after irradiation, all the samples follow a thermodynamically controlled process where they exhibit the same size, irrespective of the preparation methods, as shown in Table S2. This again suggests that the reason for the kinetically controlled step lies with the dye–dye interaction.

The size difference in different preparation methods could possibly be due to an inhomogeneous distribution of dye molecules and oppositely charged polyelectrolytes when mixed. A cooperative binding process propagates directly simultaneously with the mixing, i.e., starts before complete mixing is reached. Thus, not the equilibrium structure but a kinetically driven structure forms, which in some cases may be kinetically stabilized in its initial form. Therefore, the method of preparation influences the formation of nano-assemblies to some extent, and assemblies of desired sizes can be achieved by the corresponding choice of preparation route. The same preparation method must be followed throughout the present work to be able to understand the G5/AY38 assemblies at different charge ratios and concentrations quantitatively. Here, method I provides the most ideal mixing conditions as it leads to a thermodynamically controlled equilibrium structure; based on the above experiments, this preparation route will not be influenced by kinetic effects, as compared to methods II and III, and yield comparatively small sizes and highly stable assemblies.

### 3.3. ζ-Potential Studies

**Figure 4:**
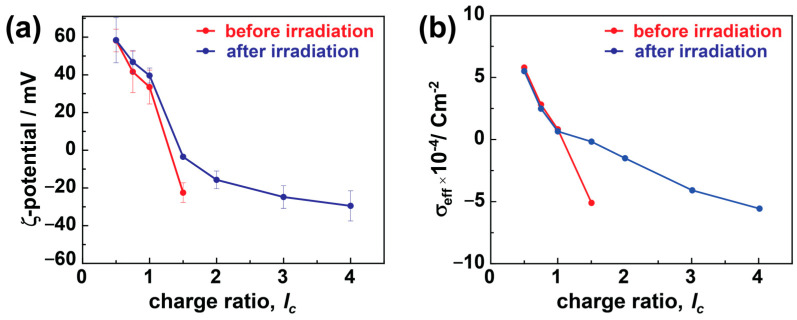
Charge characteristics of dendrimer-dye assemblies: (**a**) ζ-potential, and (**b**) effective charge density, σ_eff_. Red: before irradiation, blue: after irradiation.

The charges from dye and dendrimer play a critical role in stabilizing these electrostatic assemblies. Therefore, it is crucial to understand the diversity in particle charges due to charge ratio and irradiation and how it affects particle stability. ζ-potential studies can provide this information by measuring the electrostatic potential between the shear plane of the nanoparticles and the liquid medium, as shown in Figure 4a. In the case of dendrimer excess, the assemblies exhibit highly positive ζ-potential values of ζ > 40 mV that do not indicate any substantial changes upon irradiation in the case of TC nanostructures. In comparison, at *l_c_* > 1.0, the number of sulfonate charges increases, providing highly negative ζ-potentials of ζ < −20 mV, which electrostatically stabilizes the assemblies very well. On irradiating these particles, the ζ-potential values weaken abruptly and become less negative or close to 0 mV.

In the case of excess dendrimers (*l_c_* < 1.0), the free positive amine charges from dendrimers stabilize the assemblies, and only minimal changes in ζ-potential have been noticed upon irradiation. The nanostructures are slightly positive at a stoichiometric ratio (*l_c_* = 1.0), and the dye molecules partially redistribute to the excess dendrimer molecules and form larger aggregates than the monomers (as in host–guest assemblies), and the ζ-potential shows a positive value [39]. A similar phenomenon applies in the case of polymer brushes, where even the slight excess of polyelectrolyte charges ensures stability and forms thermodynamically controlled products. More details on the formation mechanism will be discussed in conjunction with the UV-Vis results below.

Conversely, in the case of excess dye molecules, the free sulfonate charges provide the negative ζ-potential, stabilizing the electrostatic assemblies at higher charge ratios (*l_c_* > 1). The surplus dye molecules are either present as free dye ions or bound over-stoichiometrically by dye–dye interactions with the electrostatically bound dye ions in the assembly. The surplus dye ions help stabilize the particles by providing sufficient charges to the dye–dendrimer assemblies (also the cause of negative ζ-potential, Figure 2a). At the same time, the dye–dye interactions lead to large-size aggregates and high polydispersity in the assemblies. A higher negative ζ-potential results in more densely charged and small stable assemblies along with micrometer-sized aggregates, which increase the number of large assemblies upon increasing the charge ratio. In the case of bimodal distribution (before irradiation at *l_c_* ≥ 2.0), the ζ-potential values represent the average of the different species; therefore, they cannot be interpreted much further [64]. The ζ-potential values change significantly upon irradiation in these cases due to the over-stoichiometric dye ions bound by dipole–dipole interactions. These loosely bound dye ions partially dissociate from the (bimodal) assemblies and reduce the stability of charged stabilized particles. Therefore, the weakening of the ζ-potential (increase to 0 mV) was observed upon irradiation. At the same time, these partially dissociated aggregates recombine to overcome the stability threshold and form more monodisperse and large particles that require a certain charge density for stabilization.

Considering the effective surface charge density (*σ_eff_*) is a more efficient way to understand the effects of size and charges on the particle stability. *σ_eff_* is calculated by combining the values of ζ-potential and R_H_ together by using the given formula:

(21) $$\sigma_{eff}=\frac{Q_{eff}}{S_{assembly}}=\frac{4\pi \varepsilon \zeta R_{eff}}{4\pi R_{eff}^{2}}=\frac{\varepsilon \zeta }{R_{eff}}\approx \frac{\varepsilon \zeta }{R_{H}}$$

where *Q_eff_* is the effective charge, *S_assembly_* is the assembly surface, ε is the permittivity constant, and *R_eff_* is the effective radius approximated to be the *R_H_*_._ Figure 4b plots the change in *σ_eff_* as a function of charge ratio. Prior to irradiation, the charge density decreases (from positive to negative) as the *l_c_* value increases. This is due to the increasing amount of dye compared to dendrimer, which increases the charge density of the aggregates, as discussed for the ζ-potential. *σ_eff_* is calculated only for monomodal aggregate samples. Upon irradiation, the charge density of the dendrimer excess particles nearly stays constant, further elucidating that the isomerization from *trans* to *cis* does not influence the stability as there are no dye–dye interactions present, and the assemblies are stabilized by the positive charges provided by the polyelectrolytes. In the case of moderate dye excess (*l_c_* = 1.5), the dipole–dipole bound excess dye ions (partly) dissociate upon irradiation. Thus, the small nano-assemblies are not electrostatically stabilized anymore, and larger aggregates form. Upon increasing the *l_c_* further (*l_c_* > 1.5), the previous polydispersed assemblies become monodisperse and are stabilized by their high charge density.

In summary, both the excess polyelectrolyte and dye charges can stabilize the assemblies. The nanostructures do not change upon irradiation at an excess of dendrimer charges, as the isomerization does not influence the overall charge density [39]. The dye–dye interaction between the smaller assemblies leads to larger aggregates and polydispersity in excess of anionic charges. Upon irradiation, the dye–dye interaction is interrupted, and monodisperse assemblies are formed, which are stabilized by the anionic charges.

### 3.4. UV-Vis Spectroscopy

**Figure 5:**
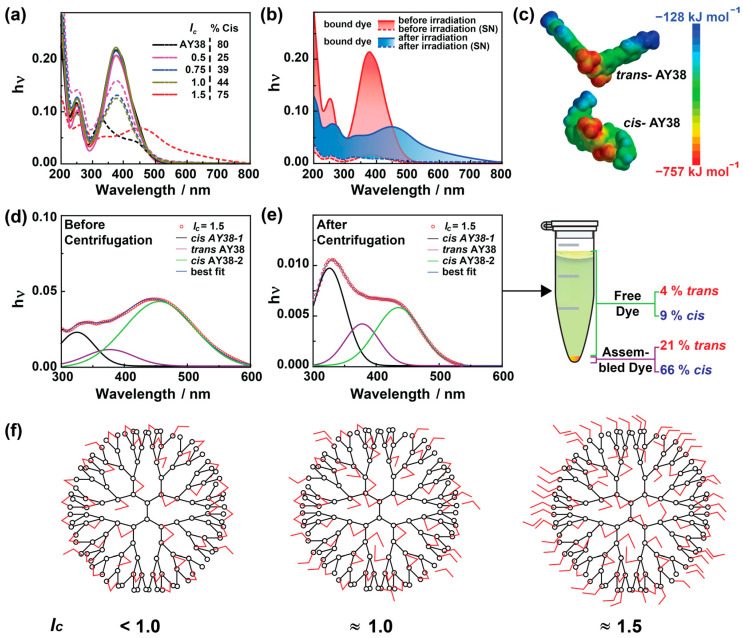
(**a**) UV-Vis spectroscopy of AY38/G5 assemblies at various charge ratios (straight lines represent the samples before irradiation, and short-dashed lines the samples after the irradiation). (**b**) The effect of centrifugation before and after irradiation (straight lines represent the samples before centrifugation and short-dashed lines the supernatant after the centrifugation. The colored area represents the dye–dendrimer assemblies). (**c**) Dipole moment of *trans* and *cis* AY38 at the molecular surface. (**d**) Analysis of UV spectra before and after irradiation, and (**e**) the supernatant after centrifugation (data are fitted using the Gauss model for multiple peaks from *cis* and *trans* AY38). The centrifuge tube represents the irradiated sample after centrifugation. (**f**) The schematic diagram represents the effect of charge ratio (*l_c_* < 1.0, *l_c_* ≈ 1.0, *l_c_* ≈ 1.5) on dye–dendrimer bindings. G4 PAMAM dendrimer is displayed as an example. Red objects designate *trans* AY38 dye molecules.

To understand the origin of the secondary dye–dye interaction, which is responsible for stabilization as well as the bimodality observed in DLS/SANS, UV-Vis measurements were performed. To compare all results, a concentration of c_AY38_ =1.0 × 10^−4^ mol L^−^ was used throughout the experiments. As shown in Figure 5a, no difference is observed in UV-Vis spectra of AY38 dye only and the solutions with dendrimer. Similar absorptions were observed before irradiation for different charge ratios, suggesting that the dendrimer addition does not affect the azo dye spectrum. This leads to the conclusion that the dye–dye interaction cannot be π–π interaction. The occurrence of hydrogen bonding can be excluded, as the dye AY38 cannot form hydrogen bonds with itself. Lastly, the hydrophobic effect can also be excluded, as the building blocks are polar molecules. Thus, it must be dipole–dipole interactions that bind multiple dye molecules to the electrostatically attached dye–dendrimer assemblies and create large aggregates. Upon irradiation, these surplus dye molecules disconnect due to *cis* isomer conformation, which is unsuitable for adjacent dipole–dipole interactions and has less than half of the dipole moment value (12.96 Debye) compared to *trans* dye molecules (27.96 Debye), as shown in Figure 5c.

To gain more information regarding stabilization and nanostructure, the amount of both *trans* and *cis* isomers was calculated prior to irradiation using the UV-Vis data fitted with Gaussian plots in Origin 9.0 software, as shown in Figure S7a. As the thermodynamically preferred molecular form, *trans* isomers remain in the majority (>90%) and do not vary upon changing the *l_c_.* However, upon irradiation, the dye isomerizes to different extents depending on the charge ratios. In dendrimer excess (*l_c_* = 0.5), only 25% of the dye converts into *cis* molecules upon irradiation, and the slightest change in size is observed. In this case, the photoprotective effect limits the dye isomerization to the *cis* state: when the dye is electrostatically bound with both sulfonate groups to the dendrimer, isomerization is hindered. With an increasing charge ratio, the degree of isomerization increases to a maximum of 75% at a moderate excess of dye (*l_c_* = 1.5), where the most noticeable difference in size is observed (Figure 2a). This increase is due to the increasing amount of dye. Due to that, likely, a lower number of dye molecules bind with both sulfonate groups to the dendrimer and are hindered from isomerizing. Upon increasing the charge ratio further, the *cis* isomer amount decreases again (Table S3). Due to the combination of electrostatic and dipole–dipole interaction, the molecule is again hindered from isomerizing. With an increasing charge ratio, the dye–dye interaction also increases, and thus the isomerization decreases.

To understand the role of both isomers in influencing the nanostructures, centrifugation was applied to the dye–dendrimer samples to quantify the *trans* and *cis* amounts in assemblies and the solution. The nanoparticles were separated from the solution using the centrifuge at the speed of 10,000 rpm for 15 min. This way, the assemblies were separated at the bottom, and only the free dye molecules remained in the supernatant (also checked by DLS, as shown in Figure S8). UV-Vis spectroscopy was then used to determine the concentration of free dye molecules in the solution after centrifugation, as shown in Figure 5b. In the case of low *l_c_* < 1, no free dye was observed in the solution as expected. The dye ions are preferably available for electrostatic interactions. Due to the strength of electrostatic interactions, the photoprotective effect occurs, limiting the dye to isomerizing in abundance (Figure 5f). The dye binds electrostatically to most dendrimer charges at a balanced *l_c_* (≈1.0). Due to the steric hindrance, a few dye molecules (≈5%) do not interact with the polyelectrolyte dendrimer but are present in the solution as a free dye (Figure S9). Due to the surplus dye ions in the solution, the calculated *l_c_* (≈0.95) is smaller than the original *l_c_* (≈1.0). Therefore, only a few charges remain in the assembly, leading to less stabilized particles and less densely charged large aggregates (up to 1 μm). After irradiation, the bound dye only isomerizes to a limited extent due to the photoprotective effect, and no significant change in the assemblies was observed.

UV-Vis absorption before and after irradiation (solid lines) is shown in Figure 5b for *l_c_* = 1.5 (concentration of AY38 = 1.0 × 10^−4^ mol L^−1^) and compares the results with the supernatant after centrifugation (short-dashed lines). The area covered by the red and blue colors represents the area occupied by dye–dendrimer assemblies; the rest belongs to the individual building blocks (free azo dye, in this case). As seen in the centrifuge tube shown in Figure 5e, the total free dye contributes approximately 13% of the total solution (a similar amount is observed before and after irradiation). As already known, the *trans* state is a more stable form of Acid Yellow 38, and the major part of the dye exists in its *trans* form (>90%) in solution before irradiation (Figure S7). After irradiation, approximately 75% of the dye isomerizes to the *cis* state, of which 9% are present as the free dye molecules. The remaining (66%) are bound in dye–dendrimer assemblies after irradiation. From 25% of the *trans* amount, 4% is present as free dye, whereas 21% contributes to the assemblies (Figure 5c–d). This suggests that due to the photoprotective effect, a portion of the *trans* molecules (25%) does not isomerize, and primarily, the free and over stoichiometric *trans* dye molecules isomerize to a *cis* state. To understand the origin of this behavior, one may consider the difference in polarity. Both isomers have different polarities due to their geometry (Figure 5c), which may cause the *cis* form to interact less strongly with other azo molecules through secondary dipole–dipole interactions.

At *l_c_* = 1.5, a high degree of isomerization is possible due to the difference in binding behavior of the two azo dye isomers. Approximately 87% of the dye is bound to the dendrimer molecules (the calculated value of *l_c_* ≈ 1.3). This must be a combination of electrostatic and dipole–dipole interactions, as shown in Figure 5f. The majority of dye ions are bound by only one site and could easily be isomerized without being influenced via the photoprotective effect.

To conclude the results from UV-Vis studies, dipole–dipole interactions are evidently involved in the structure determination and also cause the formation of polydispersed assemblies if present in excess. The photoprotective effect limits the isomerization of dye molecules and controls the size of the assemblies before and after the irradiation.

### 3.5. Isothermal Titration Calorimetry (ITC)

**Figure 6:**
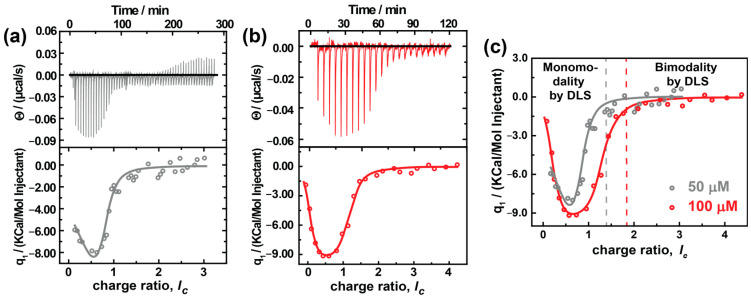
ITC analysis of AY38/G5 system using two different concentrations of AY38; (**a**) 0.5 × 10^−4^ mol L^−1^, (**b**) 1.0 × 10^−4^ mol L^−1^, and (**c**) ITC data fit comparison of two concentrations (AY38 is titrated into G5). Fits to the data according to a two-site model are shown as grey and red lines.

ITC is an excellent pathway to develop a fundamental understanding of thermodynamic parameters involved in assembly formation. Figure 6 shows the titration of different concentrations of AY38 ((0.5 and 1.0) × 10^−4^ mol L^−1^) into (1.0 and 2.0) × 10^−8^ mol L^−1^ G5 dendrimer solution. Both sets of raw data can be fitted with a two-set-of-site binding model. The fitted values are given in Table 2.

It becomes evident that the first binding step is endothermic and entropically driven with a binding enthalpy of ΔH_1,50_ = 19.9 kJ/mol and ΔH_1,100_ = 5.4 kJ/mol, respectively, and an entropy of ΔS_1,50_ = 1.3 kJ/mol and ΔS_1,100_ = 6.7 kJ/mol, respectively. This corresponds to the electrostatic interaction between the dye and the dendrimer: First, the Cl¯ counterions (from DCl) are exchanged with the -SO3¯ ions (from the azo dye), i.e., chloride ions and the hydration shell are separated from the cationic macroion charges, which are endothermic and accompanied by an increase in entropy. Contrary to the simple anticipations (where electrostatic interaction is exothermic), the enthalpy here increases. In electrostatic interactions, it may seem evident that dye ions binding to macroions will liberate energy due to the Screened Coulomb attraction between macroions and counterions. However, the exchange of counterions with its entropic effects carries the association forward, as was described for linear polyelectrolytes by Antonietti et al. [65], since dendrimers are strongly hydrophilic molecules. At the beginning of the titration, the dye ions break the hydration shell surrounding the dendrimer building blocks and remove the initial counterions. The absorption of Ca^2+^ and La^3+^ to bilayer membranes and a few colloidal objects (such as cationic micelles and liposomes) follows a similar behavior [66,67]. The difference in the enthalpy and entropy between both experiments can be understood with the concentration. At higher concentrations, the change in entropy is lower compared to lower concentrations due to the high ionic strength. Subsequently, less energy is needed to break the hydration shell. Moreover, in dilute solutions, the assemblies have more freedom of motion, which would increase the randomness and, therefore, the entropy of the solution.

Further comparing both experiments, the binding stoichiometry differs strongly. In the first step, 70 dendrimer sites become attached to dye ions electrostatically at lower concentration, but only 51 sites at higher concentration. This decrease is possibly due to the presence of a higher number of monomeric dendrimers at higher concentrations that compete for the same amount of dye-loaded dendrimers and result in a limited growth of the aggregates, as explained earlier in context with the SANS results.

As mentioned above, a two-set-of-site process is observed. The second binding step is enthalpically driven with a binding enthalpy of ΔH_2,50_ = −136 kJ/mol and ΔH_2,100_ = −34.6 kJ/mol and an entropy of ΔS_2,50_ = −101 kJ/mol and ΔS_2,100_ = −24.9 kJ/mol. The second step ranges from 0.65 ≤ *l_c_* ≤ 4.0, corresponding to the combination of electrostatic and secondary interactions. This is further evident from the binding stoichiometry, which has a value of around 220 doubly-charged dye molecules per dendrimer, while the dendrimer has only 254 charges in total. No further heat release can be observed in the ITC from *l_c_* = 1.5 onwards and *l_c_* = 2.0 onwards in the cases of c_AY38_ = 0.5 × 10^−4^ mol L^−1^ and c_AY38_ = 1.0 × 10^−4^ mol L^−1^, respectively. This is where the dipole–dipole interactions dominate over electrostatic interactions, leading to a second type of aggregation (bimodal distribution) in both concentrations, also observed in light and neutron scattering techniques. Interestingly, comparing both concentrations, the binding stoichiometry and the free energy are both the same, while the enthalpy and entropy differ. This behavior may again be due to the presence of fewer monomers at lower concentrations. As a result, the secondary interaction is stronger, and thus the enthalpy is more negative. At the same time, the entropy decreases as we observe fewer assemblies with a larger size.

Previous studies have already established that *trans* dye molecules have a higher ability to bind over-stoichiometrically to the electrostatic self-assemblies than the *cis* molecules since *cis* isomers show almost no dye–dye interaction compared to the *trans* isomers, explained by steric hindrance or geometric constraints of the *cis* dye molecules, as outlined above [13]. As shown herein, the difference in dipole moment is also an essential factor. In that study, only one binding site was observed, as Willerich et al. titrated PAMAM dendrimer into the AY38 dye solution. The only step is a combination of dye–dendrimer electrostatic interactions and dye–dye secondary interactions [14,25]. While the value for enthalpy and entropy differs from the sum observed with our system, the dye was also bound over-stoichiometrically. The reason for the differences in values is the different dendrimer generations and concentrations. In particular, the difference in generation influences (i) the electrostatic interaction, i.e., the equilibrium thermodynamics, (ii) steric effects, and (iii) the kinetics of the assembly formation. Additionally, in equilibrium, the assembly sizes increased with increasing the dendrimer generations in the previous system where the dye undergoes dye–dye interactions [68]. This is in accordance with the study of Willerich et al., where they observed a more negative ΔG of the association while forming larger assemblies [15]. However, the overall results of the two studies are consistent with a more detailed study and the interpretation provided herein. A detailed study of the generation dependency and its effects on electrostatic assemblies will be addressed in a future study.

Overall, ITC confirms that the dipole–dipole interaction causes the polydispersity of the nano-assemblies. The changes observed in DLS and SANS regarding the different concentrations can also be observed and understood with ITC. At the same time, while it is clear that the concentration influences the size, the overall thermodynamic behavior is the same for both concentrations.

### 3.6. General Discussion

**Figure 7:**
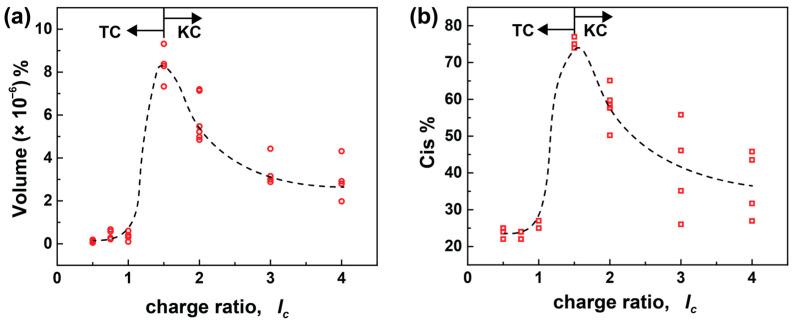
The influence of the charge ratio on (**a**) change in the volume % and (**b**) total *Cis* % for the AY38/G5 system upon irradiation using multiple concentrations (c_AY38_ = 0.5 × 10^−4^ mol L^−1^, c_AY38_ = 1.0 × 10^−4^ mol L^−1^, c_AY38_ = 1.5× 10^−4^ mol L^−1^, c_AY38_ = 2.0 × 10^−4^ mol L^−1^). R_H_ values were obtained from multiangle DLS and converted into the volume using the formula of a sphere volume (V = $\frac{4}{3}$πR_H_^3^), and the *cis* % was calculated using UV-Vis spectroscopy (see experimental pasrt). The arrows represent the starting point of the TC and KC processes. The open symbols represent samples from different concentrations at the same charge ratio.

From the above experiments, it is confirmed that the secondary interactions that come into effect once the ionic building blocks approach each other ultimately affect the assembly formation processes. To further understand the role of secondary interactions in detail on kinetically and thermodynamically controlled assemblies, the irradiation-caused change in particle volume in % and the total amount of *cis* dye in % are plotted against the *l_c_* of the sample (Figure 7). The change in volume % and the degree of isomerization due to irradiation share a similar dependency on the charge ratio. Due to the majority of small aggregates in bimodal systems, only the small particles are considered in the plot above. The values in Figure 6 are taken from 0.5 ≤ *l_c_* ≤ 4.0 with multiple concentrations (c_AY38_ = (0.5, 1.0, 1.5, and 2.0) × 10^−4^ mol L^−1^). The volume % and the *cis* % increase up to the TC process (≤1.5) and start declining when KC assemblies form.

At an excess of dendrimer charges, no significant changes in the volume and isomerization upon irradiation take place. Due to the photoprotective effect, nearly no isomerization occurs. Therefore, no change is observed upon irradiation and the particles are stabilized by the surplus dendrimer charges. Nevertheless, the samples containing a moderate excess of dye (*l_c_* = 1.5) show a sharp increase in size and *cis* % due to their unique combination of electrostatic and secondary interactions, as discussed above. On further increasing the dye amount, the excess of dye ions assists in stabilizing the assemblies, leading to smaller percentage changes in particle volume and amount of *cis* dye, as compared to the moderate excess of dye. Moreover, the secondary interactions dominate at high *l_c_* and cause the formation of (kinetically controlled) bimodal aggregates. Consequently, the *cis* amount directly affects the size of the aggregates. Hence, the combination of charge ratio and concentration allows control of the degree of isomerization and, ultimately, the size and structure of the nano-assemblies.

## 4. Conclusions

Insight has been gained in controllably fabricating functional nanostructures using electrostatic self-assembly in an aqueous environment. This study presented the facile route to regulate the aggregation of small building blocks and create nanoscale structures with diverse shapes and sizes. The electrostatic assemblies of generation 5 PAMAM dendrimer and AY38 azo dye have been investigated, where the effects of charge ratio and concentration have been observed, including the quantitative analysis of kinetically and thermodynamically controlled assemblies. Monodispersed particles with narrow size distributions and varying shapes are primarily achieved in either samples with dendrimer excess or samples with a moderate excess of dye (*l_c_* ≤ 1.5). The surplus charges (positive and negative) help in stabilizing the assemblies in the solution. In contrast, bimodal and multimodal distributions were found in the samples of higher *l_c_* (>1.5). Remarkably, upon irradiation, the structure of the nano-assemblies changes to well-defined monodispersed assemblies and also to polydispersed nano-assemblies.

Further, the changes in size upon irradiation have been directly correlated with the percentage of *cis* dye, since the *cis* isomer cannot undergo dipole–dipole interactions. The concentration was further used to manipulate the size and shape of the assemblies. The size decreased with increasing the concentrations of building blocks while keeping the charge ratio constant. This type of secondary interaction has been defined as dipole–dipole interaction. In the future, varying the dendrimer generation could be valuable here as other generations provide different numbers of charges and additional flexibility in the molecule, which may influence the polydispersity. The generation dependency will be addressed in a future publication.

## Figures and Tables

**Table 1:** Nanoparticle shapes of AY38/G5 nanoparticles at different charge ratios obtained by SLS-SANS experiments (size is not up to the scale). The green and magenta colors represent samples at C_AY38_ = 0.5 × 10^−4^ mol L^−1^ before and after irradiation. The red and blue color show the samples at C_AY38_ = 1.0 × 10^−4^ mol L^−1^ before and after irradiation.

*l_c_*	C_AY38_ = 0.5 × 10^−4^ mol L^−1^		C_AY38_ = 1.0 × 10^−4^ mol L^−1^	
	Before Irradiation	After Irradiation	Before Irradiation	After Irradiation
0.5	Shape Variation in AY38/G5 Assemblies		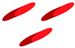	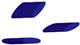
1.5			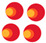	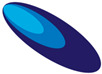
2.0	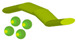	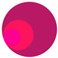	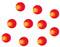	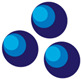
3.0	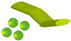	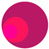	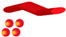	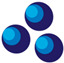
4.0	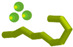	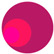	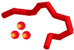	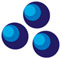

**Table 2:** ITC results of AY38/G5 system. AY38 is titrated into G5. Two-site model: (a) ΔH: 3–5%; (b) K: 20–60%; (c) Dye ions/den: 5–10%.

C_AY38_ /(×10^−4^) mol L^−1^	Dye Ions/Den	K_1_/M^−1^	ΔH_1_ per Site /kJ mol^−1^	ΔS_1_ per Site /J mol^−1^K^−1^	ΔG_1_ per Site /kJ mol^−1^	Dye Ions/Den	K_2_/M^−1^	ΔH_2_ per Site /kJ mol^−1^	ΔS_2_ per Site /J mol^−1^K^−1^	ΔG_2_ per Site /kJ mol^−1^
0.5	70	2.2 × 10^10^	19.9	5.4	−0.86	222	1.0 × 10^13^	−136	−101	−0.33
1.0	51	4.5 × 10^9^	6.7	1.3	−1.1	216	1.1 × 10^13^	−34.6	−24.9	−0.34

## Data Availability

The data presented are inclided in this study. SANS-data will be available at doi:10.5291/ILL-DATA.9-12-662 and doi:10.5291/ILL-DATA.9-12-624].

## Supplementary Materials

The following supporting information can be downloaded at: https://www.mdpi.com/article/10.3390/polym16010050/s1. Figure S1: Dynamic light scattering of AY38/G5 assemblies; Figure S2: Dynamic light scattering of AY38/G5 assemblies at charge ratio lc = 30.0 before irradiation; Figure S3: Thermodynamically and kinetically controlled assemblies; Figure S4: SLS-SANS of AY38/G5 assemblies at different charge; Figure S5: Electric-field autocorrelation function g1(τ) and distribution of relaxation times A(τ) (scattering angle θ = 90°) of AY38/G5 assemblies after irradiation at lc = 0.5 (thermodynamically controlled), and 2.0 (kinetically controlled; Figure S6: SLS-SANS of AY38/G5 at lc = 2.0 using two preparation routes and the effect of irradiation on assemblies; Figure S7: Centrifugation: Analysis of UV-Vis spectra before irradiation for AY38/G5 at lc = 1.5; Figure S8: Electric field autocorrelation function g1(τ) for the supernatant received after centrifugation of lc = 1.5; Figure S9: The effect of centrifugation on the absorbance of AY38/G5 at lc = 1.0 is recorded by UV-Vis studies; Table S1: Radius of gyration for multiple charge ratios at different concentrations using SANS models and the Guinier; Table S2: DLS table for AY38/G5 using different methods of preparation; Table S3: Bound cis% in the assemblies after irradiation by varying the concentration and charge ratio of AY38/G5 assemblies.
