# Supplementary material for: Controlling the Morphology in Electrostatic Self-Assembly via Light
Source: Polymers (Basel). 2023 Dec 22;16(1):50. doi: 10.3390/polym16010050 (PMC10780651; doi:10.3390/polym16010050)
Supplement: Supplementary file 1 [file polymers-16-00050-s001.zip › polymers-2750237-supplementary.pdf]

Supporting Information

Results:

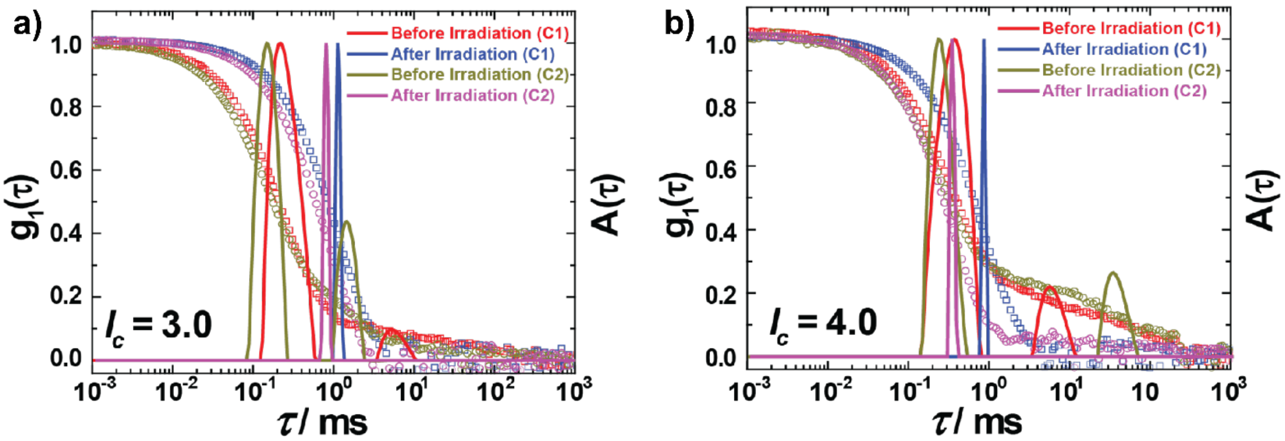

Figure S1 Dynamic light scattering of AY38/G5 assemblies at charge ratio I_c =3.0 **(a)** and 4.0 **(b)** before and after UV irradiation for concentration C1 = 0.5 × 10^{-4} mol L^{-1} AY38 and C2 = 1.0 × 10^{-4} mol L^{-1} AY38: Electric field autocorrelation function $g_1(\tau)$ and distribution of relaxation times $A(\tau)$ at a scattering angle of θ = 90 show the increase in assembly size. R_H = hydrodynamic radius; σ = standard deviation of the distribution.

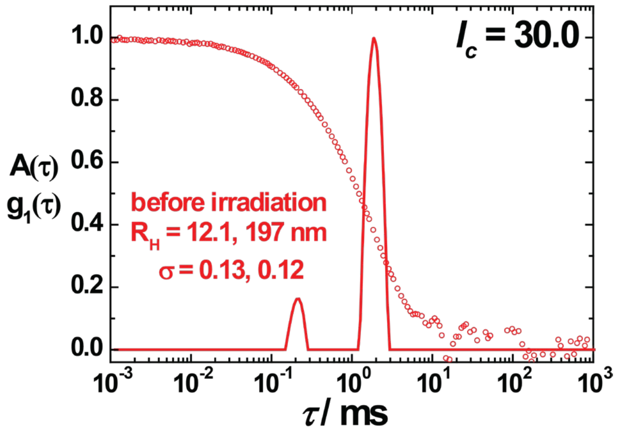

Figure S2 Dynamic light scattering of AY38/G5 assemblies at charge ratio I_c = 30.0 before irradiation at C_{AY38} = 1.0 × 10^{-4} mol L^{-1}.T

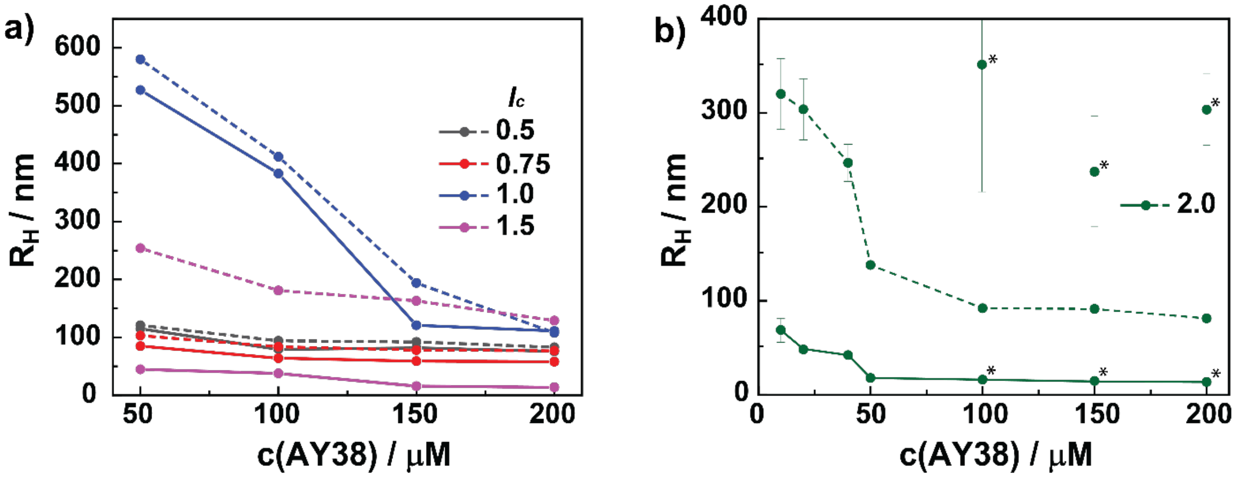

Figure S3 Various samples are divided into **(a)** Thermodynamically controlled and **(b)** Kinetically controlled assemblies based on their charge ratios, concentration, and the effect of irradiation. The straight line represents samples prior to irradiation, and the dashed lines after irradiation. Stars in **(b)** are used for samples with a bimodal distribution **1** Radius of gyration for multiple charge ratios at different concentrations using SANS models and the Guinier approximation. The error is < 10% in all the cases (Concentration is given in terms of AY38).

Table S1 Radius of gyration for multiple charge ratios at different concentrations using SANS structural models and the Guinier approximation to check the correctness of the fitted model. The error is < 10% in all the cases (Concentration is given in terms of AY38).

**Charge Ratio**	**1.5**	**2.0**	**2.0**	**3.0**	**3.0**	**4.0**	**4.0**
**AY38 Concentration / μM**	**100**	**50**	**100**	**50**	**100**	**50**	**100**
				**R_G / nm**			
**Before irradiation(exp)**	35.9		7.6				
**Before Irradiation(Calc)**	21.6		7.9				
**After Irradiation(exp)**	107.8	81.2	54.1	61	32.4	77	36.5
**After Irradiation(Calc)**	99.3	77	56.7	62.3	32.6	76	33.9

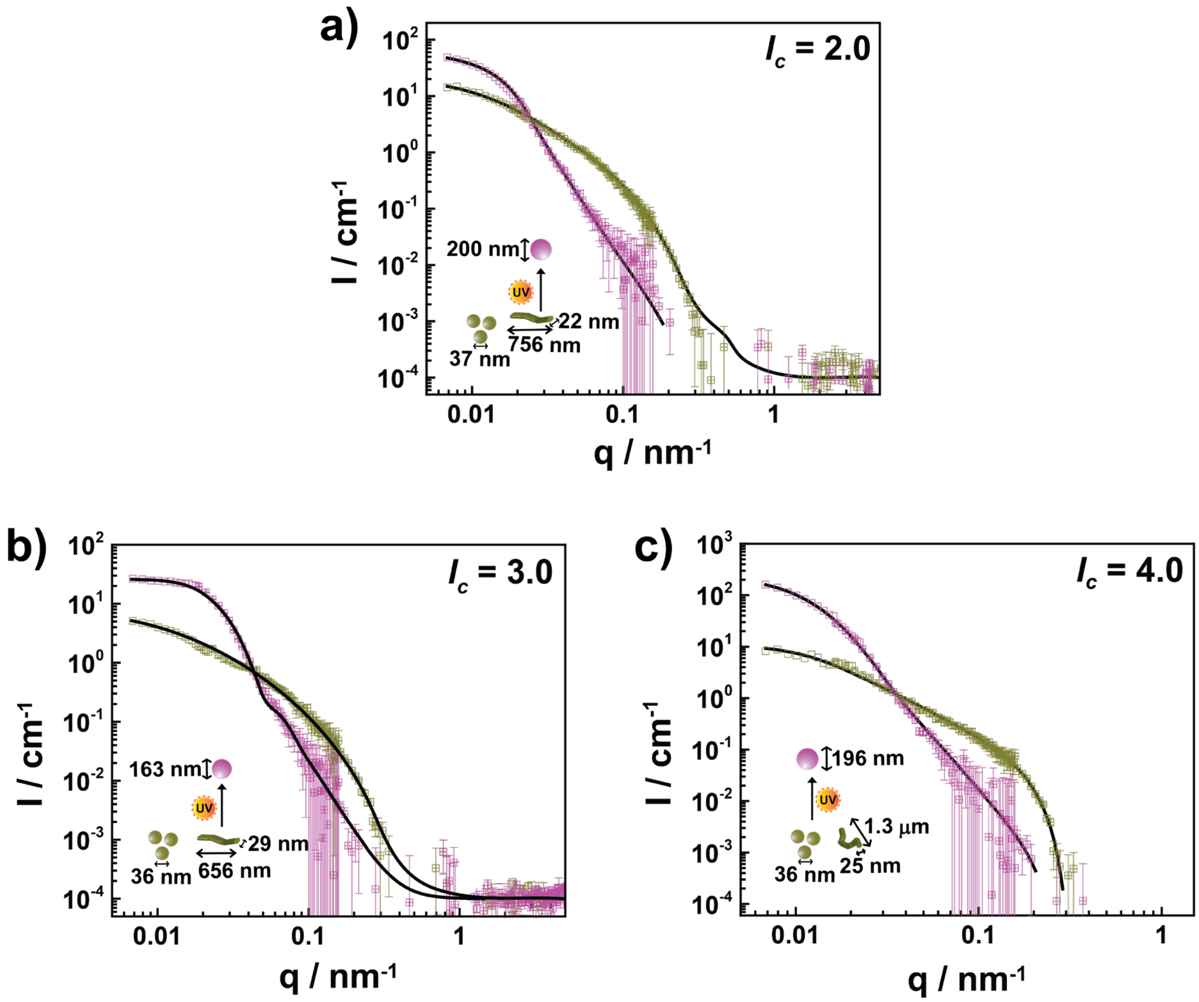

Figure S4 SLS-SANS of AY38/G5 assemblies at charge ratios l_c: **(a)** 2.0; **(b)** 3.0; and **(c)** 4.0. Dark yellow and magenta color represent the nanoparticles before and after irradiation, black straight lines represent the best fit. (C_{AY38} = 0.5 × 10^{-4} mol L^{-1}). The shapes shown in the figures are obtained by fitting the SLS-SANS data using SasView 4.2.2 software. The sizes of the figures are not up to the scale.

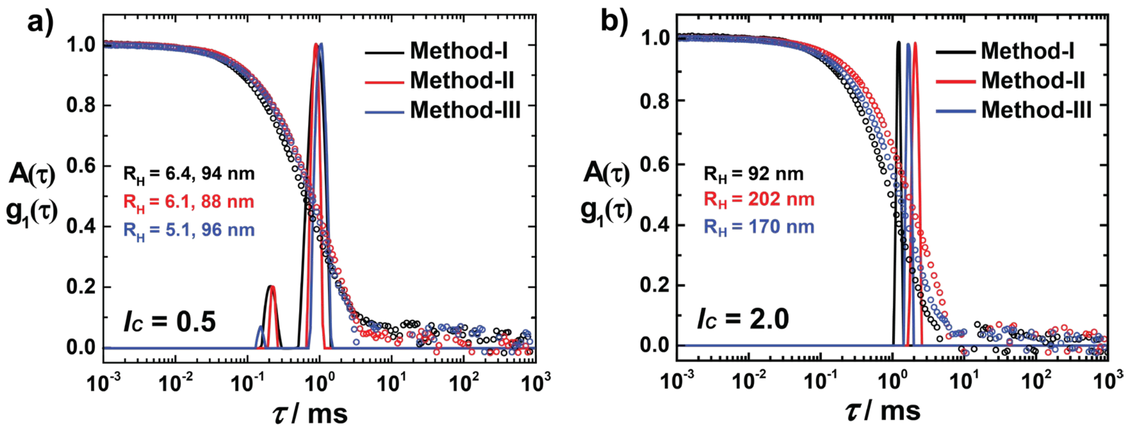

Figure S5 Electric-field autocorrelation function $g_1(\tau)$ and distribution of relaxation times $A(\tau)$ (scattering angle θ = 90°) of AY38/G5 assemblies after irradiation at I_c = **(a)** 0.5 (Thermodynamically controlled), and **(b)** 2.0 (Kinetically controlled), where, method-I is the preparation way used throughout this study. In method-II and III, stock solutions were prepared and mixed in acidic medium (pD = 3.5). In method II, dendrimer solution was added to the dye solution, while in method III, it was the other way around.

Different preparation routes distinguish the thermodynamic and kinetic controlled samples based on their charge ratios [1]. In method-I, the stock solutions of AY38 and G5 PAMAM dendrimer were mixed in basic (pD = 10.5) deuterated solution while continuously stirring, and 2-3 μL of 1 M DCl was added into it to change the pD to 3.5 where electrostatic assemblies started forming. Whereas, in the case of method-II and III, both the stock solutions were mixed in an acidic solution while stirring, in which AY38 dye was added first in method II and G5 was added first in method III. The dynamic light scattering results of I_c = 0.5 and 2.0 are given below, with the R_H values prepared by all three methods mentioned above in Figure S5.

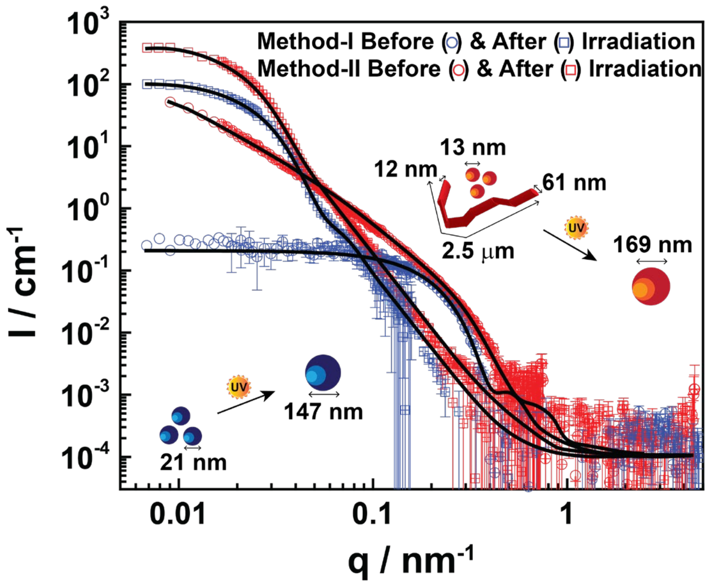

Figure S6 SLS-SANS of AY38/G5 assemblies at l_c = 2.0 using two preparation routes and the effect of irradiation on assemblies. Blue and red color represents method I and II, where open circles show the samples before irradiation and open squares after irradiation. The black line shows the best fit for the data displayed.

Figure S6 shows the SLS-SANS result of AY38/G5 at l_c = 2.0, where two preparation routes are used to formulate the assemblies. Both methods result in different structures before irradiation. Using method-I, we obtain monomodal particles with spherical form factor whereas, in method-II, similar small spheres form along with elongated flexible cylindrical structures up to 2.5 μm in size, which, upon irradiation, converts into large and highly monodisperse spherical particles in both cases.

Table S2 DLS for AY38/G5 system using different methods of preparation. In method-I, all the building blocks were prepared and mixed in acidic medium. In method II, the stock solutions were prepared at *pD* = 10.5, and later, DCl was added to initiate the assemblies. R_{H1} and R_{H2} represent the bimodality.

AY38/G5	Before Irradiation				After Irradiation	
	Method-I		Method-II		Method-I	Method-II
I_c	R_{H1} / nm	R_{H2} / nm	R_{H1} / nm	R_{H2} / nm	R_H / nm	R_H / nm
**0.5**	89 ± 8		86 ± 11		94 ± 5	93 ± 6
**0.75**	64 ± 4		60 ± 9		84 ± 14	86 ± 9
**1**	383 ± 38		366 ± 43		412 ± 34	440 ± 29
**1.5**	38 ± 3		19 ± 5	147 ± 23	181 ± 12	205 ± 16
**2**	18 ± 2	350 ± 140	29 ± 3	914 ± 324	92 ± 3	224 ± 11
**3**	13 ± 1	362 ± 181	10 ± 1	242 ± 103	54 ± 1	280 ± 15
**4**	12 ± 1	1550 ± 1100	12 ± 1	552 ± 215	47 ± 1	145 ± 7

Table S3 shows the percentage of isomerization upon irradiation at different charge ratios and concentrations. The dye concentration does not seem to affect the *cis* % in low to moderate I_c (≤ 1.5), but it starts to decrease by increasing the concentration in case of dye excess.

AY38/μM	0.5<br>Cis%	0.75<br>Cis%	1.0<br>Cis%	1.5<br>Cis%	2.0<br>Cis%	3.0<br>Cis%	4.0<br>Cis%
**20**					65		
**40**					60		
**50**	22	22	27	77	58	56	46
**100**	25	24	27	75	58	46	44
**150**	24	22	27	74	57	35	32
**200**	25	24	25	74	50	26	27

In Figure S7, the amount of dye isomers was calculated before and after centrifugation at I_c = 1.5 in AY38/G5 assemblies. Prior to irradiation, the more stable *trans* molecules were

present in the majority (> 90%), leaving < 10% of cis dye molecules. The data shown in Figure S7 is fitted by multiple peak fit using the Gaussian model in Origin software. The change of isomers amount can be noticed in Figure 5(d-e), where, after irradiation, the *cis* molecules are in the majority.

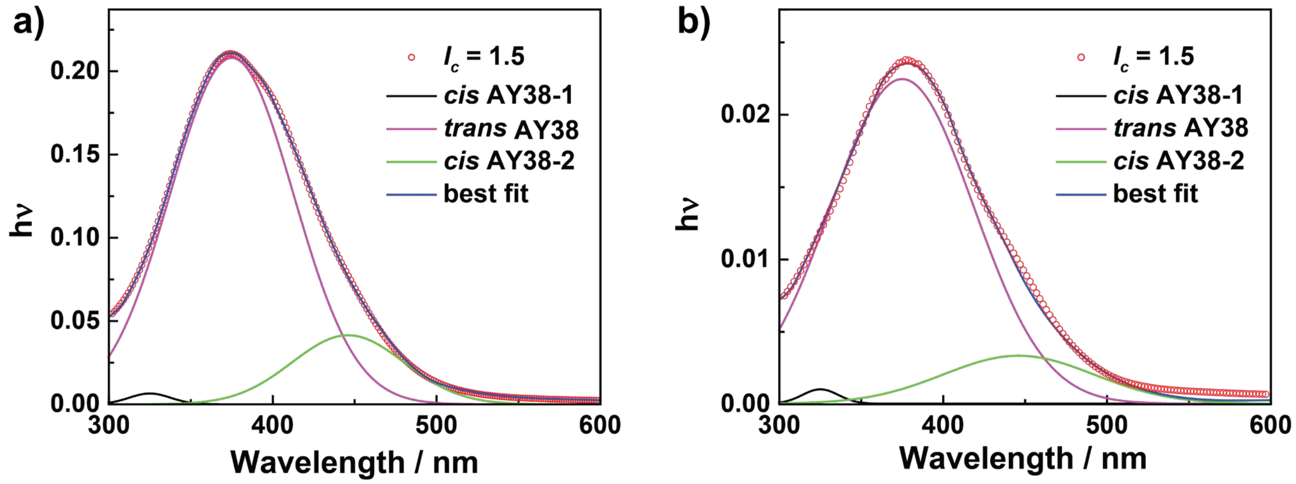

Figure S7 Analysis of UV-Vis spectra before irradiation for AY38/G5 at I_c = 1.5 for (d) before centrifugation and (e) supernatant after centrifugation. (Data is fitted using the Gauss model for multiple peaks from cis and trans-AY38. The centrifuge tube represents the irradiated sample after centrifugation.)

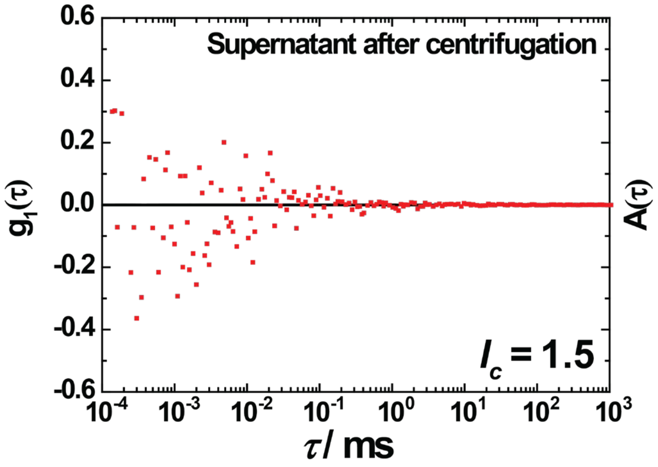

Figure S8 Electric field autocorrelation function $g_1(\tau)$ at a scattering angle of τ = 90° for the supernatant received after centrifugation of I_c = 1.5 prior to irradiation.

No particles were observed from the supernatant, as can be seen from the DLS result of AY38/G5 assemblies at I_c = 1.5 in Figure S8 after centrifuging the assemblies using the ultracentrifuge for 15 min at the speed of 10000 rpm.

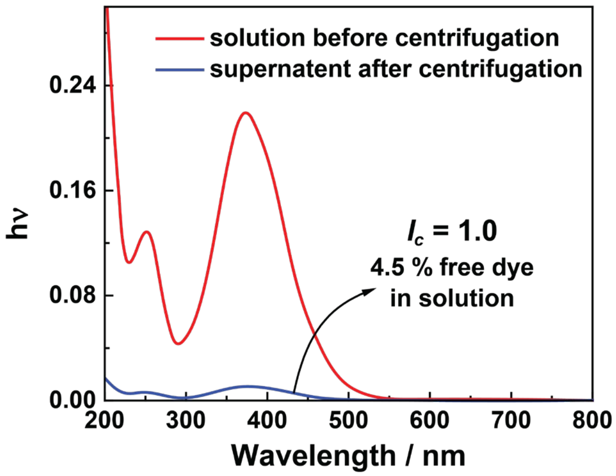

Figure S9 The effect of centrifugation on the absorbance of AY38/G5 at I_c = 1.0 is recorded by UV-Vis studies.

In the case of I_c = 1.0, approximately 4.5% of the free dye is available in the solution, which ultimately decreases the value of the original I_c from 1.0 to 0.95. Due to this, the assemblies are not entirely neutral at the balanced charge ratio and show positive charges in ζ-potential studies.

References:

1. Willerich, I.; Li, Y.; Gröhn, F. Influencing Particle Size and Stability of Ionic Dendrimer-Dye Assemblies. *J. Phys. Chem. B* **2010**, *114*, 15466–15476, doi:10.1021/jp107358q.

2. Willerich, I.; Gröhn, F. Thermodynamics of Photoresponsive Polyelectrolyte-Dye Assemblies with Irradiation Wavelength Triggered Particle Size. *Macromolecules* **2011**, *44*, 4452–4461, doi:10.1021/ma200538e.
